# Early Multidisciplinary Rehabilitation Improves Swallowing and Speech Function in a Patient With Amyotrophic Lateral Sclerosis

**DOI:** 10.1002/ccr3.71486

**Published:** 2025-11-16

**Authors:** Yue Hu, Yun Lu, Danmei Lan

**Affiliations:** ^1^ Department of Neurology and Neurological Rehabilitation Shanghai Yangzhi Rehabilitation Hospital (Shanghai Sunshine Rehabilitation Center), School of Medicine, Tongji University Shanghai China

**Keywords:** amyotrophic lateral sclerosis, dysarthria, dysphagia, multidisciplinary rehabilitation, video fluoroscopic swallowing study

## Abstract

Amyotrophic lateral sclerosis (ALS) is a chronic, progressive neurodegenerative disease for which there is a lack of effective treatment. This case report describes a 49‐year‐old male with ALS who presented with dysphagia, dysarthria, dyskinesia, sleep disorders, anxiety, and depression. Following 45 days of early multidisciplinary rehabilitation, the patient demonstrated significant improvement in swallowing and speech function, alleviation of non‐motor symptoms, and maintenance of motor function. Notably, he retained the ability to consume soft foods at a two‐year follow‐up. This case highlights the vital role of early multidisciplinary rehabilitation in the comprehensive management of ALS.

**Summary**

- This study examines early multidisciplinary rehabilitation in ALS management.
- A 49‐year‐old male with ALS showed improved swallowing, speech, and non‐motor symptoms after 45 days of treatment, with sustained motor function.
- Two‐year follow‐up revealed preserved soft‐food intake, highlighting the benefits of early intervention.

## 1 Introduction

Amyotrophic lateral sclerosis (ALS) is a chronic, progressive degenerative disease of the nervous system [1]. Most patients die within 3–5 years of diagnosis due to complications such as respiratory failure, lung infections, and pressure sores [2]. Currently, no curative treatments exist, making the management of ALS focused on alleviating functional impairments, enhancing quality of life, and prolonging survival.

ALS manifests through diverse motor impairments (limb weakness, bulbar and respiratory dysfunction) and non‐motor symptoms (sleep, mood, cognitive disturbances) [3]. The American Academy of Neurology recommends early referral to multidisciplinary clinics for comprehensive care [4]. These clinics integrate neurology, rehabilitation medicine, physical/occupational/speech therapy, nutrition, psychiatry, and social work to address current needs while providing anticipatory guidance for disease progression [5]. This collaborative approach ensures coordinated management throughout the disease course.

A recent meta‐analysis of 13 RCTs demonstrated that rehabilitation therapies significantly improve Amyotrophic Lateral Sclerosis Functional Rating Scale Revised (ALSFRS‐R) scores and respiratory function in ALS patients [6]. Complementary studies highlight specific benefits: perceptual learning aids speech recognition [7], and biofeedback training mitigates swallowing‐related decline [8]. Multidisciplinary care is consistently linked to prolonged survival, improved quality of life, and better management of disease complications [9, 10].

Aligned with this evidence, we present a case of a 49‐year‐old male with ALS exhibiting dysphagia, dysarthria, and neuropsychiatric symptoms. Following 45 days of comprehensive multidisciplinary rehabilitation, the patient exhibited marked improvements in swallowing and speech, alleviation of non‐motor symptoms, and maintenance of motor function. This case reinforces the critical value of integrated, early rehabilitation and underscores its potential to improve core functional outcomes in ALS.

Ethics approval was granted by the Medical Ethics Committee of Shanghai Yangzhi Rehabilitation Hospital (No. 2023‐007), and written informed consent was obtained from the participant.

## 2 Case Presentation

### 2.1 Presenting Complaints and Disease History

A 49‐year‐old male was diagnosed with ALS one year prior, following a two‐year history of progressive limb weakness, atrophy, and fasciculations. Over the past six months, he developed worsening dysarthria, dysphagia, and frequent choking. He was non‐ambulatory and fully dependent for daily activities. Associated symptoms included weight loss of 4.5 kg, constipation, poor sleep, and decreased appetite. There was no relevant family history.

Neurological examination revealed dysarthria, mild cognitive impairment, and tongue fasciculations. Motor findings included limb atrophy, generalized weakness (muscle strength grade 2–3), and hypertonia. Reflexes were hyperactive with bilateral ankle clonus, and positive Hoffman, palmar, and Babinski signs.

Needle electromyography revealed widespread active denervation (fibrillation potentials and positive sharp waves) with chronic neurogenic changes (enlarged MUPs) in cranial, cervical, thoracic and lumbosacral regions. Nerve conduction studies, including F‐wave analysis, remained within normal limits. Neuroimaging of the neuraxis ruled out structural lesions. Comprehensive laboratory studies, including CSF analysis, onconeural antibodies, and genetic testing for ALS‐related genes, excluded alternative diagnoses.

### 2.2 Multidisciplinary Comprehensive Assessment

The patient's status was assessed across multiple domains by experienced clinicians within one week of admission, using a comprehensive battery of validated scales (Initial evaluation, Table 1).

**TABLE 1:** Comparison of assessment before and after rehabilitation treatment.

	Initial evaluation	Final evaluation
ALSFRS‐R	24	25
BBS	26	26
MBI	32	32
Modified Frenchay Scale	129	132
ECAS	79	82
HAMA	11	9
HAMD	22	15
FSS	44	42
VAS	5	5
FVC (%)	66	70
AHI	14.2	5.8
PSQI	13	10

Abbreviations: AHI, Apnea Hypopnea Index; ALSFRS‐R, Amyotrophic Lateral Sclerosis Functional Rating Scale—Revised; BBS, Berg Balance Scale; ECAS, Edinburgh Cognitive and Behavioral ALS Screen; FSS, Fatigue Severity Scale; FVC, Forced Vital Capacity; HAMA, Hamilton Depression Scale; HAMD, Hamilton Anxiety Scale; MBI, Modified Barthel Index; PSQI, Pittsburgh Sleep Quality Index; VAS, Visual Analogue Scale.

#### 2.2.1 Motor and Functional Assessment Scales

The patient's functional status was assessed at admission using standardized scales. The ALSFRSR (0‐48) measured global function, with higher scores indicating greater independence [11]. The Berg Balance Scale (BBS, 0–56) evaluated balance and fall risk [12]. The Modified Barthel Index (MBI, 0–100) quantified independence in activities of daily living [13]. Swallowing and speech function were assessed with the Modified Frenchay Scale [14]. A Video Fluoroscopic Swallowing Study (VFSS) was performed, revealing delayed swallow initiation, penetration, and aspiration with watery foods (Video 1), as well as prolonged oral transit and residue with pudding‐like consistencies (Video 2).

**VIDEO 1:** The video of the patient drinking watery food before treatment. Video content can be viewed at https://onlinelibrary.wiley.com/doi/10.1002/ccr3.71486.

**VIDEO 2:** The video of the patient eating pudding‐like food before treatment. Video content can be viewed at https://onlinelibrary.wiley.com/doi/10.1002/ccr3.71486.

#### 2.2.2 Cognitive and Neuropsychological Assessment Scales

Cognitive and behavioral functions were screened using the Edinburgh Cognitive and Behavioral ALS Screen (ECAS, 0‐136), where a higher total score indicates better function [15]. Anxiety and depressive symptoms were clinically rated with the Hamilton Anxiety Scale (HAMA, 0–56) and the 17‐item Hamilton Depression Scale (HAMD, 0–52), with higher scores denoting greater severity [16, 17]. The Fatigue Severity Scale (FSS, 1–7) quantified fatigue impact, and a Visual Analogue Scale (VAS, 0–10) evaluated the intensity of subjective symptoms like pain [18, 19].

#### 2.2.3 Respiratory Function and Sleep Assessment Measures

Spiratory function was assessed by the percentage of predicted Forced Vital Capacity (FVC%), with a higher value indicating better function [20]. The severity of sleep‐disordered breathing was measured by the Apnea‐Hypopnea Index (AHI) via polysomnography, where a higher index signifies worse apnea [21]. Subjective sleep quality was evaluated using the Pittsburgh Sleep Quality Index (PSQI, global score 0–21), with a higher score denoting poorer sleep quality [22].

### 2.3 Therapeutic Intervention

A 6‐week multidisciplinary rehabilitation program was tailored to address the patient's functional impairments. Pharmacological management included riluzole for disease progression modification, neuroprotective agents, and mosapride citrate to address gastrointestinal motility. Nutritional support provided a high‐protein, finely chopped diet based on VFSS findings, while respiratory management involved nocturnal non‐invasive ventilation for sleep apnea.

The comprehensive exercise regimen incorporated daily stretching and joint mobility training (10 min), bilateral lower limb strength training (2 sets of 12 repetitions), upright balance practice (5 min), and MotoMed resistance training for both upper and lower limbs (20 min). Cardiopulmonary rehabilitation included thoracic expansion exercises and incentive spirometry training (20 min daily). Activities of daily living training encompassed balance transfer exercises, upper limb functional training, and self‐care practice including feeding and dressing tasks.

Speech and swallowing rehabilitation comprised respiratory support training, oromotor exercises targeting cheek muscles, lips, and tongue movement, along with soft palate elevation techniques. Additional interventions included speech volume and rhythm training through poetry recitation and singing, as well as specific tongue motor training incorporating the Mendelssohn maneuver and effortful swallowing techniques.

The daily three‐hour training was segmented with rest periods and individually titrated based on fatigue scales and vital signs. Adjunctive therapies included weekly psychotherapy, 20 sessions of low‐frequency rTMS to the DLPFC, and monthly hyperbaric oxygen therapy (0.18 MPa, 60 min/session, 25 sessions).

### 2.4 Outcomes and Follow‐Up

**FIGURE 1:**
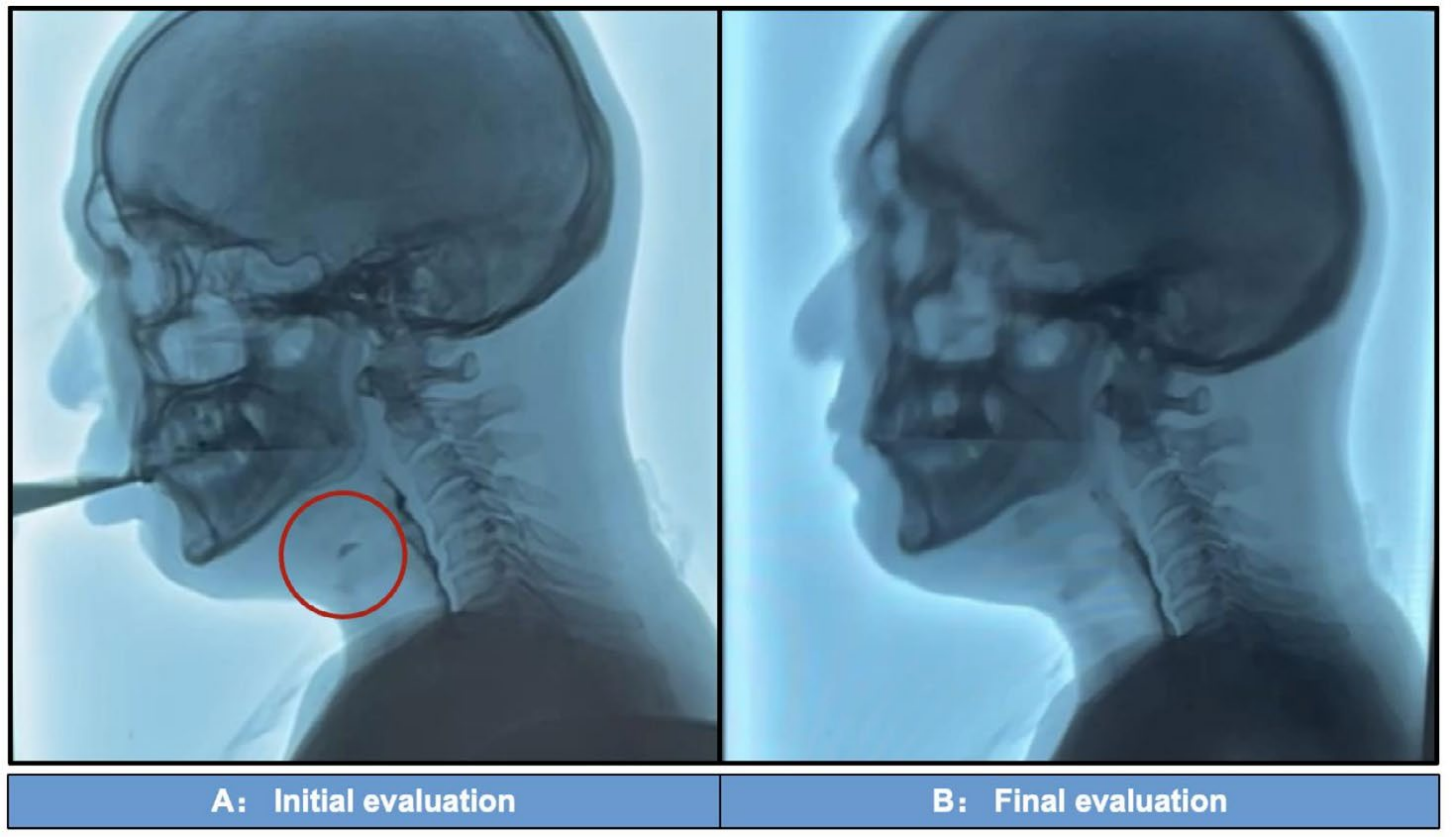
Comparison of video fluoroscopic swallowing study results before and after rehabilitation. (A) Obvious aspiration before treatment, with the red circle indicating aspiration; (B) No asphyxia, no aspiration, no penetration, no residual asphyxia after 45 days of rehabilitation; soft food intake could be completed.

After 45 days of treatment, the rehabilitation assessment was performed again before discharge (Final evaluation, Table 1). Swallowing safety improved substantially, with VFSS confirming elimination of penetration and aspiration during watery foods ingestion (Video 3) and significantly enhanced tongue coordination during pudding‐like thick food consumption (Video 4). The comparison of watery food before and after treatment is shown in Figure 1. Speech production improved through better respiratory support and vocal control. Motor function showed bilateral improvement in proximal limb control.

**VIDEO 3:** The video of the patient drinking watery food after treatment. Video content can be viewed at https://onlinelibrary.wiley.com/doi/10.1002/ccr3.71486.

**VIDEO 4:** The video of the patient eating pudding‐like food after treatment. Video content can be viewed at https://onlinelibrary.wiley.com/doi/10.1002/ccr3.71486.

At the two‐year follow‐up without continued rehabilitation, the patient maintained soft diet tolerance but experienced substantial speech deterioration, precluding verbal communication. The ALSFRS‐R score declined to 14/48, indicating progressive functional decline despite previous gains.

## 3 Discussion

ALS is a fatal neurodegenerative disease involving upper and lower motor neurons, leading to progressive muscle weakness, atrophy, and spasticity. These impairments often result in malnutrition and aspiration risk, significantly increasing mortality [23]. This case demonstrates that early multidisciplinary rehabilitation can markedly improve bulbar function and maintain motor capabilities in ALS patients.

Studies confirm that swallowing abnormalities, particularly pharyngeal‐phase aspiration with liquids, are common in ALS patients even without overt dysphagia complaints [24, 25]. Early dysphagia primarily manifests orally [26], with tongue dysmotility being a key contributor, leading to oropharyngeal residue [24]. In managing our patient with choking symptoms, we implemented a finely chopped diet, targeted swallowing training, and postural adjustments (chin tuck). These interventions resulted in safe oral intake without aspiration or residue on follow‐up VFSS, enabling maintenance of nutritional status with soft, high‐calorie foods [27].

Dysarthria universally affects ALS patients, progressing from reduced speech intelligibility to complete anarthria. Although evidence for speech rehabilitation remains limited, our patient achieved measurable gains in articulatory control through systematic vocalization training, suggesting early intervention may optimize residual bulbar function. For advanced cases, augmentative and alternative communication systems—including emerging brain‐computer interface technologies—provide essential communication support [28, 29, 30].

Current evidence supports moderate‐intensity exercise for ALS patients, as it may slow functional decline and improve motor performance [31, 32], while excessive training can be detrimental [33, 34]. Our bedridden patient received daily stretching to prevent contractures. Exercise prescription requires careful monitoring, with training modification indicated if post‐exertional fatigue persists beyond 30 min.

This patient presented with respiratory impairment (FVC 66% predicted, AHI 14.2, nocturnal SpO_2_ 86%). Following daytime cardiopulmonary training with nocturnal non‐invasive ventilation, respiratory function improved (FVC 70%, AHI 5.8). This aligns with evidence supporting non‐invasive ventilation for ALS and respiratory training for functional enhancement [35]. While hyperbaric oxygen therapy was included to address tissue hypoxia, its efficacy in ALS requires further investigation.

Sleep disorders affect approximately 70% of ALS patients, while anxiety and depression occur in about 14% and 7% of cases, respectively [36, 37]. For our patient with mild‐to‐moderate anxiety, depression, and poor sleep quality, we administered cognitive‐behavioral therapy and repetitive transcranial magnetic stimulation (rTMS) targeting the right dorsolateral prefrontal cortex—an evidence‐based approach recommended for depressed patients with comorbid sleep disturbances [38]. Following this intervention, the patient demonstrated noticeable improvements in both mood and sleep quality. Worsening psychiatric symptoms would necessitate pharmacologic management under psychiatric supervision.

This study's primary limitation lies in determining the specific contribution of each intervention within our multidisciplinary approach. While the concurrent administration of riluzole, rTMS, hyperbaric oxygen, and targeted rehabilitation precludes isolating individual effects, several factors suggest task‐specific training played a central role. The significant bulbar improvements are unlikely attributable to riluzole, which primarily slows progression rather than restoring function. Although rTMS improved non‐motor symptoms and hyperbaric oxygen may have enhanced exercise tolerance, their direct impact on swallowing and speech remains unestablished. Thus, the functional gains likely resulted primarily from neuromuscular adaptation through repetitive task‐oriented training, though synergistic effects cannot be excluded. Future controlled trials should investigate the efficacy of individual components and their potential interactions.

## 4 Conclusion

For ALS patients, multidisciplinary rehabilitation teams are essential to develop personalized interventions that alleviate symptoms, maximize residual function, and enhance social participation. Such comprehensive care reduces caregiver dependence and improves quality of life. However, current rehabilitation protocols require further validation through large‐scale, high‐quality clinical trials to establish evidence‐based practice standards.

## Author Contributions

**Yue Hu:** conceptualization, data curation, formal analysis, investigation, writing – original draft. **Yun Lu:** data curation, writing – review and editing. **Danmei Lan:** conceptualization, data curation, formal analysis, funding acquisition, investigation, methodology, supervision, writing – review and editing.

## Ethics Statement

The authors have nothing to report.

## Consent

Written, informed consent was obtained from the patient for publication of this case report and the accompanying images.

## Conflicts of Interest

The authors declare no conflicts of interest.

## Data Availability

All data analyzed in this case report is included in the article. No additional datasets are available.
